# Should I stay or should I go: TFIIIC as assembly factor and barrier in RNA polymerase III transcription

**DOI:** 10.1042/BST20253058

**Published:** 2025-08-05

**Authors:** Wolfram Seifert-Davila, Maria Elize van Breugel, Fred van Leeuwen, Christoph W. Müller

**Affiliations:** 1 Molecular Systems Biology Unit, European Molecular Biology Laboratory (EMBL), Heidelberg 69117, Germany; 2 Division of Gene Regulation, Netherlands Cancer Institute, Amsterdam 1066 CX, Netherlands; 3 Department of Medical Biology, Amsterdam UMC, University of Amsterdam, Amsterdam 1105 AZ, Netherlands

**Keywords:** general transcription factor, RNA polymerase III, TFIIIC, transcription initiation, tRNA transcription

## Abstract

Critical for the regulation of eukaryotic gene transcription is the assembly and interplay of general transcription factors (GTFs) with RNA polymerases (RNAPs), leading to the formation of pre-initiation complexes (PICs) as a rate-limiting step in transcription activation. Compared with RNAPII PIC assembly involving many GTFs, activators, and co-activators, RNAPIII PIC assembly is less complex, involving mainly the four GTFs TFIIIA, TFIIIB, TFIIIC, and snRNA activating protein complex with only a few additional factors. The RNAPIII-specific GTF TFIIIC is present in type I and II promoters. One prominent area of investigation has been the dynamic interaction between TFIIIC and its promoter elements, the varying affinities of TFIIIC toward these elements, and the flexible linker within TFIIIC. Additionally, evidence suggests that TFIIIC may play a dual role, acting as an assembly factor that positions TFIIIB during PIC formation and as a barrier during RNAPIII-mediated transcription. By summarizing recent structural, biochemical, and genomic data, this review explores the mechanisms by which RNAPIII-specific GTFs, with a focus on TFIIIC, dynamically regulate RNAPIII transcription.

## Introduction

Transcription by RNA polymerase III (RNAPIII) requires the highly co-ordinated interplay between its core machinery and a set of general transcription factors (GTFs) that recognize distinct promoters. RNAPIII transcribes small, structured, non-coding RNAs using three distinct promoters containing conserved RNAPIII-specific GTF DNA-binding sites. Type I promoters found in 5S rRNA genes contain A-box, intermediate element (IE), and C-box; type II promoters found in tRNA genes contain A-box and B-box; and type III promoters found in U6 snRNA genes contain TATA-box, proximal sequence element (PSE), and distal sequence element (DSE) (reviewed in [1]). TFIIIB is present in all three promoter types and is composed of three subunits: TFIIB-related factor 1 (Brf1), TATA-binding protein (TBP), and B-double prime 1 (Bdp1). In contrast, TFIIIA is specific to type I, TFIIIC to type I and II, and snRNA activating protein complex (SNAPc) to type III promoters in vertebrates, with each of them binding to specific DNA sequences [2]. Type I and type II promoters contain discontinuous GTF binding sequences located within the transcribed genes, referred to as internal control regions. In contrast, in type III promoters, SNAPc binding sites are exclusively located in the 5′-flanking region (reviewed in [3]).

In this review, we will summarize how the RNAPIII GTFs are assembled on type I, II, and III promoters. Subsequently, we will focus specifically on the six-subunit GTF TFIIIC, which is proposed to have multiple roles in the cell. In particular, we will discuss recent insights into the engagement of yeast and human TFIIIC on type I and II promoters and its role in RNAPIII transcription initiation, as well as the proposed dual role of TFIIIC as an assembly factor and barrier of transcription depending on cellular growth conditions.

## Transcription from type I promoters

**Figure 1:**
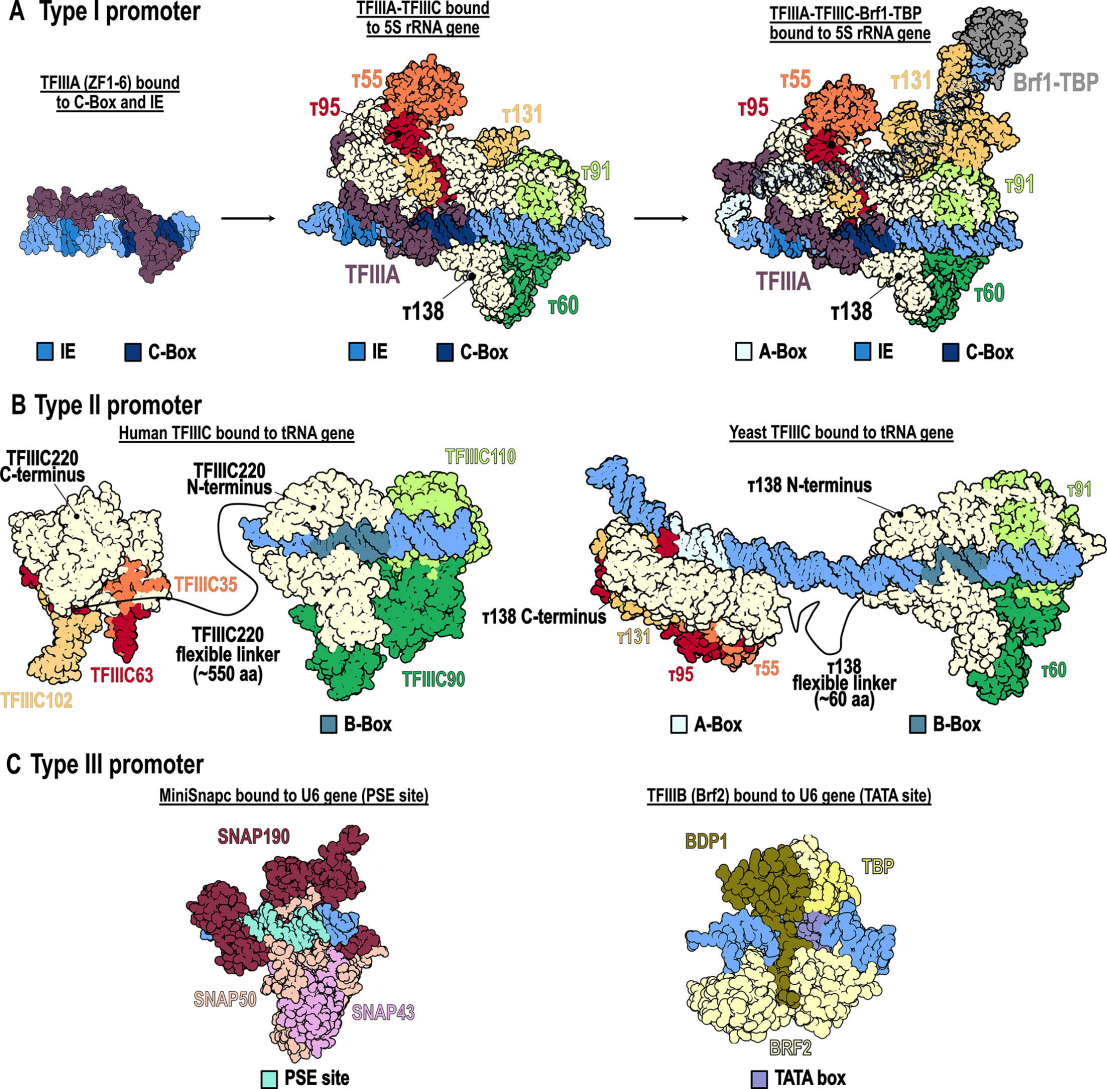
Available crystal and cryo-EM structures of individual RNAPIII GTFs and complexes bound to their promoter elements. (**A**) **Type I promoter**. TFIIIA (ZF1–ZF6) bound to the C-box and IE (PDB: 1TF6); cryo-EM structure of TFIIIA-TFIIIC bound to the 5S RNA gene, where PDB:8FFZ was fitted into the corresponding density map (EMD-29358) with unsupported regions removed; cryo-EM structure of TFIIIA-TFIIIC-Brf1-TBP bound to the 5S RNA gene, which wraps around the complex (PDB:8FFZ). (**B**) **Type II promoter**. Left panel: Cryo-EM structures of human TFIIIC bound to a tRNA gene include the unbound τA subcomplex (PDB:8CLK) and the τB subcomplex bound to the B-box (PDB:8CLI). Right panel: Fully engaged yeast TFIIIC bound to a tRNA gene is represented by cryo-EM structures of the τA subcomplex (PDB:9GCK) and the τB subcomplex (PDB:9GC3). So far, no structural information about a TFIIIC-TFIIIB complex has been reported. The complete yeast TFIIIC–DNA model was built by positioning a 45-bp tRNA^His^ DNA duplex into the τA–DNA map and a 40-bp segment into the τB–DNA map, with orientations and distances refined using cryo-EM single-particle mapping described in [5]. (**C**) **Type III promoter**. Cryo-EM structure of miniSNAPc bound to the PSE motif (PDB: 7XUR); crystal structure of TFIIIB (Brf2 type) bound to its TATA box site (PDB: 5N9G).

For type I promoters, the GTF TFIIIA contains nine zinc fingers (ZF) and recognizes the A-box with low affinity, whereas it binds to the IE and C-box with higher affinity [4]. The crystal structure of *Xenopus laevis* TFIIIA (ZF1–ZF6) bound to DNA shows sequence-specific major groove interactions of ZF1–ZF3 with the C-box; ZF5 makes sequence-specific contacts with the IE in the major groove, while ZF4 and ZF6 interact with the minor groove (Figure 1A) [6].

Once TFIIIA recognizes the promoter, it facilitates the recruitment of TFIIIC, followed by TFIIIB, which subsequently leads to the recruitment of RNAPIII [2]. Cryo-EM structures of *Saccharomyces cerevisiae* TFIIIA-TFIIIC and TFIIIA-TFIIIC-Brf1-TBP complexes revealed how the type I promoter architecture is formed with the 5S rRNA gene wrapping around the TFIIIA-TFIIIC complex [7]. The TFIIIA-TFIIIC complex is stabilized by the presence of Brf1-TBP, and bending of the DNA is critical for positioning these GTFs (Figure 1A). This structural arrangement, where the 5S rRNA gene wraps around the TFIIIA-TFIIIC complex, may explain why, on average, the transcription start site (TSS) in type I genes is located 50 bp upstream from the boundary of the A-box, compared with 10 bp in type II genes, despite both gene types containing an A-box.

## Transcription from type II promoters

**Figure 2:**
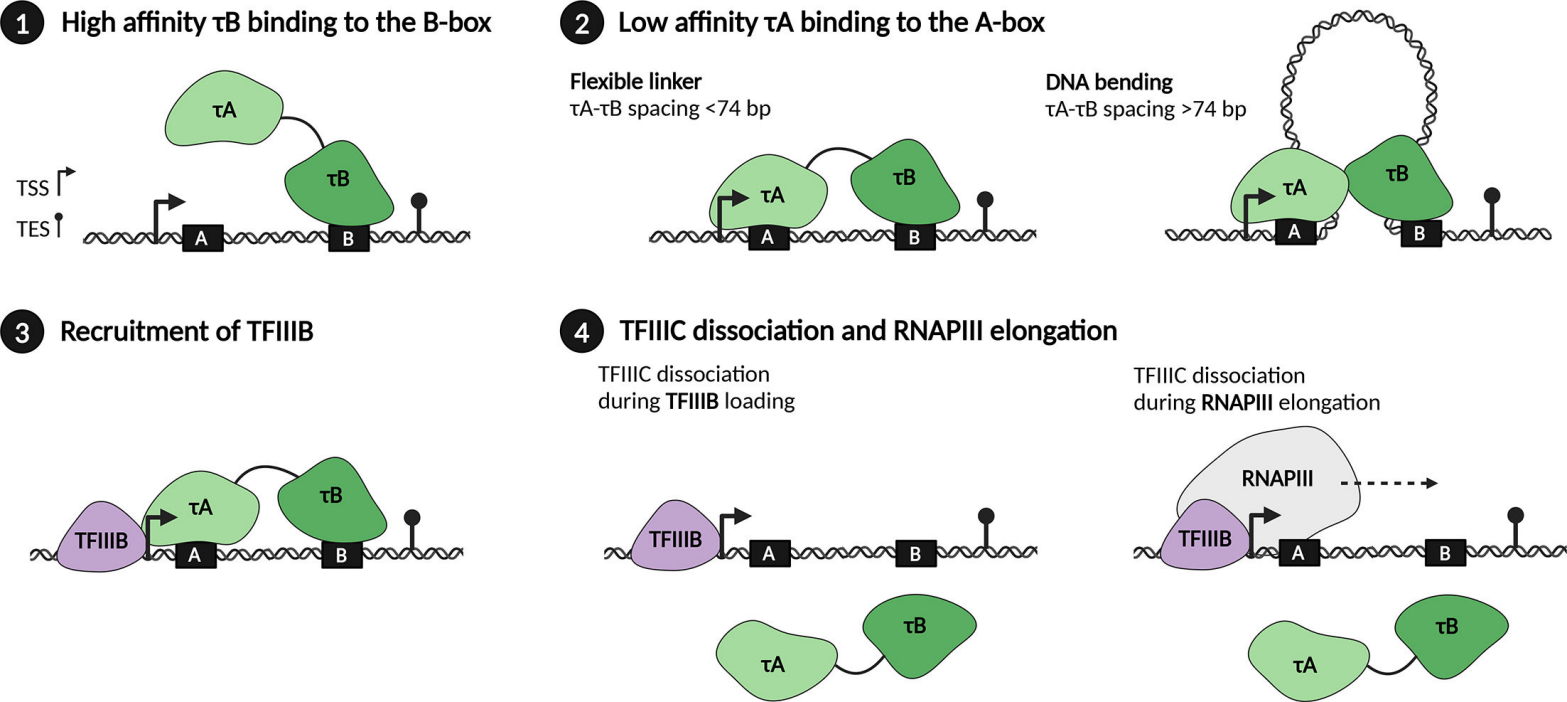
The dynamics of TFIIIC during RNAPIII transcription initiation. (**1**) The TFIIIC submodule τB binds with high affinity to the B-box and anchors to the tRNA gene. (**2**) This facilitates subsequent recruitment of τA to the A-box with weaker affinity. To bridge the region between the A- and B-box, TFIIIC can simultaneously engage with both promoter elements due to a flexible linker in subunit TFIIIC220 in human and subunit τ138 in yeast for spacings <74 bp. Subunit TFIIIC220 in human TFIIIC and yeast τ138 take part in both the τA and the τB subcomplexes, with its N-terminus belonging to τB and its C-terminus to τA. When the intervening sequence between the A- and B-box exceeds 74 bp, TFIIIC could adapt by inducing DNA looping. (**3**) Once TFIIIC is assembled on the tRNA gene, TFIIIB is recruited and placed upstream the TSS. (**4**) Whether TFIIIC is subsequently fully released from the tRNA gene remains unclear. Two hypotheses have been proposed: 1) The τA lobe gets detached upon interaction with TFIIIB or 2) interaction with RNAPIII disrupts the interaction between τA and τB to promote TFIIIC dissociation. TSS, transcription start site; TES, transcription end site.

Similar to the non-continuous intragenic promoter structure of the type I 5S rRNA gene, tRNA genes feature two distinct intragenic conserved regions: the A-box and B-box, which correspond to the D-loop and T-stem/T-loop regions of tRNAs, respectively [8]. The spacing between A- and B-boxes varies from 32 to 93 bp in *S. cerevisiae* type II promoters [5]. Scanning transmission electron microscopy suggested that six-subunit TFIIIC may utilize a flexible linker that connects τA and τB subcomplexes to simultaneously engage with the A-box and B-box [9]. The flexible linker between τA and τB has been hypothesized to accommodate A-box and B-box spacing <74 bp. For spacing exceeding 74 bp, it has been suggested that TFIIIC is able to bend DNA to bridge the distance (Figure 2). In favor of this, electron microscopy experiments have shown that TFIIIC sharply bends the DNA upon interaction [9]. In addition, genome-wide footprinting *in vivo* (‘bootprinting’) of intron-containing tRNA genes (gene sizes of 89–133 bp) showed partial protection of the intervening DNA, suggesting the formation of a DNA loop that remained unprotected [10].

Initially, the flexible linker between τA and τB was attributed to the τ131 subunit, as its tetratricopeptide repeats were thought to act as a hinge upon interaction with DNA [11]. Later, structural studies and cross-linking mass spectrometry data identified the interaction region (τIR) of yeast TFIIIC subunit τ138 as the main link between τA and τB, with adjacent disordered regions that could provide the flexibility needed to accommodate various spacings between A-box and B-box [12]. Only recently, cryo-EM structures of the complete TFIIIC complex in human [13] and yeast [5] provided further insight, revealing that the flexible linker is a disordered region approximately 550 amino acids long in the largest human subunit, TFIIIC220 (Figure 1B **, left panel**), and around 60 amino acids long in yeast subunit τ138 (Figure 1B **, right panel**). In human TFIIIC, the linker spans ~200 Å to connect τA and τB subcomplexes when τB is bound to the B-box (although τA remains unbound to its target promoter). In *S. cerevisiae,* the linker spans ~170 Å when τA and τB simultaneously engage with the A-box and B-box separated by a 32-bp intervening sequence.

## Transcription from type III promoters

In type III promoters, the promoter region is located upstream of the TSS and external to the gene being transcribed, a main distinction from type I and II promoters [2]. The U6 RNA gene from *Xenopus tropicalis* was one of the first identified type III genes, containing elements typical of RNAPII promoters, but its α-amanitin-resistant transcription confirmed RNAPIII activity [14]. Subsequent studies identified the factor binding to the PSE element, referring to it as the PSE-binding protein [15], PSE transcription factor [16], or SNAPc [17], which was subsequently confirmed to be the same complex.

The SNAPc complex consists of five subunits: SNAP190, SNAP50, SNAP45, SNAP43, and SNAP19 [18]. SNAPc has been characterized in metazoans and plants but not in yeast. Until recently, only the structure of a human miniSNAPc, consisting of SNAP190, SNAP50, and SNAP43, which is sufficient for transcription, was resolved. This structure reveals its interaction with the PSE site in a ‘wrap-around’ binding mode (Figure 1C) [19]. The promoter recognition of SNAPc begins with the weak binding of SNAPc to the PSE motif, which is subsequently stabilized by TFIIIB, specifically by TBP binding to the TATA box. This interaction is stabilized by OCT1 and ZNF143, which are engaged with the DSE promoter region [2]. In type III promoter recognition, the TFIIIB complex includes a variant subunit called BRF2 [20,21], and its interaction with the promoter region has been structurally characterized, first in a BRF2-TBP-DNA complex [22] and subsequently in a BRF2-TBP-BDP1-DNA complex [23] (Figure 1C). No structural information of a binary complex between SNAPc and TFIIIB is available so far. In contrast, structures describing complete human SNAPc-TFIIIB-RNAPIII complexes [24,25] and the transition between the human RNAPIII pre-initiation complex (PIC) and RNAPIII elongation complexes on a type III promoter [26] have been recently determined, providing insight into SNAPc-mediated RNAPIII transcription initiation and elongation.

## Engagement of TFIIIC on type II promoters

Electrophoretic mobility shift assays identified the B-box in type II promoters as the predominant TFIIIC binding site, where subcomplex τB binds with nanomolar affinity [27] compared with the A-box that is bound only with micromolar binding affinity by the τA subcomplex [28,29]. The recent cryo-EM structures of human and yeast TFIIIC have demonstrated how subcomplexes τA and τB interact with their respective DNA elements [5,13]. The τB subcomplex binds the B-box with high affinity through a combination of DNA shape and sequence recognition. In its largest subunit, human TFIIIC220 (yeast τ138), four winged-helix domains almost completely enclose the B-box, forming multiple interactions with conserved DNA bases and the DNA backbone. In addition, the heterodimer human TFIIIC110/TFIIIC90 (yeast τ91/τ60) interacts with the DNA backbone in both human and yeast. In yeast, it also makes base-specific contacts downstream of the B-box. In contrast, subcomplex τA interacts with the A-box much more transiently. In the cryo-EM structure of the human TFIIIC-DNA complex, τA is observed only in the DNA-unbound state [13], whereas, in the *S. cerevisiae* TFIIIC-DNA complex, τA interacts primarily through DNA shape recognition, including dynamic movements of its τ95-DNA-binding domain along the DNA [5]. Single-molecule fluorescence assays not only revealed that the binding of both subcomplexes occurs very fast, within 200 ms, but also confirmed that the B-box is mainly responsible for TFIIIC engagement with a TFIIIC-bound residence time *in vitro* of less than a minute [5], aligning well with live-cell imaging data [30].

## DNA rearrangements upon TFIIIC-TFIIIB complex formation

## TFIIIC during RNAPIII elongation

Upon recognizing the promoter regions of tRNA genes, TFIIIC facilitates the recruitment of TFIIIB in the context of type II promoters, thereby providing the platform for RNAPIII to initiate transcription [31]. The cryo-EM structure of TFIIIA- and TFIIIC-dependent RNAPIII transcription initiation on the type I promoter provides new insights into the role of TFIIIC-mediated RNAPIII recruitment. In the TFIIIA-TFIIIC-TBP-Brf1-DNA complex, the DNA wraps around the TFIIIA-TFIIIC complex stabilized by Brf1-TBP [7], while the N-terminal domain of yeast subunit τ131 (human TFIIIC102) undergoes a large conformational change, thereby positioning Brf1-TBP close to the TSS. A similar large conformational change of yeast subunit τ131 (human TFIIIC102) has also been suggested on type II promoters [28]. Footprinting experiments in the presence of TFIIIB and TFIIIC show a strong bend of type II promoter DNA between A-box and B-box and between A-box and TATA-like element, suggesting an important role of the A-box in selecting the TSS [5]. In contrast, detailed structural information of TFIIIC and TFIIIB bound to type II promoter DNA is still lacking, perhaps because TFIIIC subcomplexes rapidly dissociate from the promoter once TFIIIB is positioned.

Once TFIIIB is positioned upstream of the tRNA gene, it has been suggested that TFIIIC is dispensable for RNAPIII transcription, since TFIIIB alone correctly positions RNAPIII on the tRNA gene. TFIIIB is therefore proposed as the RNAPIII transcription initiation factor, while TFIIIC is an assembly factor for TFIIIB [32]. In agreement with this, TFIIIC can be selectively stripped away using heparin treatment *in vitro* once TFIIIB is assembled [32]. In addition, A-box protection was lost when RNAPIII was added in enzymatic footprinting experiments [33]. This suggests that at least τA dissociates from the tRNA gene upon RNAPIII entry. Additionally, it was found that TFIIIC is displaced from the DNA during RNAPIII transcription using an *in vitro* reconstituted system [34] and that TFIIIC is not a bona fide component of the PIC [28]. However, the situation might be more complex, as *in vitro* transcription assays also demonstrated that TFIIIC is only dispensable for the re-initiation on small RNAPIII genes (~100 bp), while TFIIIC is required for the re-initiation of longer RNAPIII genes (>300 bp) [35]. Whether TFIIIC completely dissociates from the promoter still remains unclear as ‘facilitated re-initiation,’ where RNAPIII re-engages with the same promoter and its pre-bound GTFs might benefit from TFIIIC remaining at least partially bound [36]. One hypothesis is that a region in Rpc53, a subunit of RNAPIII, which is shown to interact with τ131, promotes TFIIIC dissociation upon RNAPIII elongation [37]. On the contrary, other studies suggest that the interaction between TFIIIB and τ131 induces a conformational change which promotes TFIIIC dissociation from the DNA [7,28] (Figure 2). Therefore, it remains unclear if and at which step during PIC assembly TFIIIC dissociation takes place.

## TFIIIC as a transient barrier of RNAPIII transcription

If TFIIIC is not fully released during transcription elongation, it is suggested that TFIIIC could act as a roadblock [38]. Using nascent transcript sequencing, it was revealed that RNAPIII pauses at specific sites within tRNA genes [38]. These sites coincide with TFIIIC binding sites, and the authors hypothesize that TFIIIC may act as a physical roadblock to interfere with RNAPIII elongation and cause transient pausing. If TFIIIC could act as a roadblock for RNAPIII elongation, it could thereby potentially act as a negative regulator of RNAPIII transcription. In support of this idea, chromatin immunoprecipitation studies have shown that occupancy of RNAPIII and TFIIIC is anti-correlated in living cells [39–43]. In yeast, acute nutrient deprivation reduces RNAPIII occupancy (Rpc82 and Rpc40) within 25 minutes, while TFIIIB binding (Brf1) remains unchanged. In contrast, TFIIIC occupancy (τ95 and τ91) increases rapidly within 25 minutes after nutrient deprivation. Prolonged nutrient deprivation (75–180 minutes) causes TFIIIB to decrease 2–3-fold, similar to RNAPIII [39,41]. Additionally, genome-wide occupancy profiling during the yeast stationary phase showed that TFIIIC (τ95) remained bound, while RNAPIII occupancy (Rpo31), and to a lesser extent TFIIIB (Bdp1), decreased [40]. Although TFIIIC occupancy increases under repressive conditions, it does not reach the level of RNAPIII and TFIIIB on tRNA genes, even though their occupancy decreases. The dynamics observed for individual subunits were recently extended to the other members of the multi-subunit complexes of TFIIIC, TFIIIB, and RNAPIII [43]. The increase in TFIIIC occupancy upon nutrient stress was shown to be hampered in *maf1*Δ strains, although this effect was only significant for intron-containing tRNA genes [39]. However, these results could reflect indirect effects of the *maf1*Δ strains, since RNAPIII occupancy is drastically increased in these strains in repressive conditions, potentially blocking TFIIIC binding [39]. In contrast, genome-wide profiling of TFIIIC in *maf1*Δ strains showed relatively little effects compared with wild-type cells, although different repressive conditions were used (rapamycin treatment and starvation instead of growth in glycerol at 37°C) [44]. Whether it is the competitive binding between RNAPIII and TFIIIC, or whether an unknown factor is responsible for increased TFIIIC recruitment in repressive conditions, remains to be determined.

Additional support for the role of TFIIIC in repressive conditions comes from co-immunoprecipitation (co-IP) experiments, which showed that the interaction between Bdp1 and τ95 increases during nutrient deprivation [42]. These results were later corroborated by additional co-IP studies that showed increased interaction between Bdp1 or Brf1 and τ131 in crosslinked chromatin in a repressed state [39]. Although the interaction between TFIIIB and TFIIIC increases in repressive conditions [39,42], TFIIIB occupancy on chromatin does not increase in acute nutrient deprivation and decreases under prolonged stress [39,41]. This suggests that the dynamic interactions observed by co-IP are different from those observed in chromatin IP. Possibly, the co-IP results could be representing protein–protein interactions in the soluble state, and the Brf1 and Bdp1 interaction with TFIIIC during repression is separated from tRNA genes. Alternatively, the increase in chromatin occupancy in repressive conditions could be due to an increase in TFIIIC protein levels or activity in the nucleus, while TFIIIB protein levels and activity remain similar. However, the protein expression of TFIIIC subunits is generally not affected by repressive conditions [39,43]. Nonetheless, the full picture of the transcription factor complex dynamics in response to nutrient perturbations remains to be resolved. Further studies are needed to understand how the observed increased interaction between TFIIIB and TFIIIC at the global protein level under repressive conditions relates to the activity and binding of the protein complexes to specific tRNA genes.

In contrast with the proposed repressive role of TFIIIC, TFIIIC is also known to interact with factors that could relieve RNAPIII repression. For example, in murine fibroblasts and HeLa cells, TFIIIC is found to be associated with the mammalian target of rapamycin (mTOR) through the TOS motif in TFIIIC63 [45]. It is hypothesized that TFIIIC recruits mTOR to RNAPIII-transcribed genes, which stimulates RNAPIII transcription by phosphorylation and inactivation of Maf1 [45]. The interaction with activating factors like mTOR suggests that TFIIIC functions differently in active and repressive conditions, corroborating the dynamic behavior described previously.

**Figure 3:**
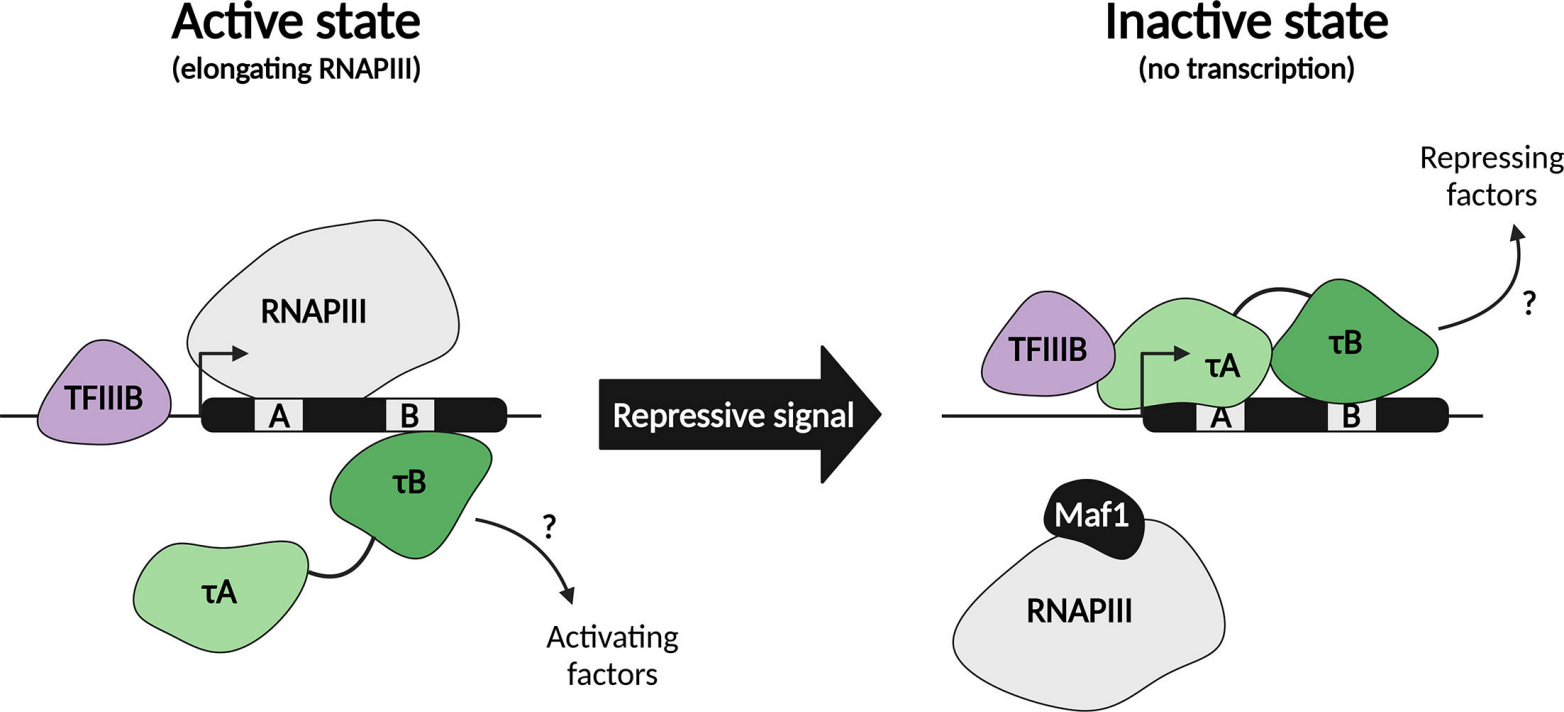
A two-state model for TFIIIC. In the active state (left), when RNAPIII elongation takes place, at least τA dissociates from the tRNA gene, which allows RNAPIII to move along the tRNA gene to perform transcription elongation. Possibly, τB still interacts with the tRNA gene to interfere with elongating RNAPIII by acting as a roadblock. Upon repressive signals (right), RNAPIII occupancy at the tRNA is decreased and is bound by Maf1. In addition, TFIIIC occupancy on the chromatin is increased. The interaction between TFIIIC and TFIIIB is increased, and possibly TFIIIB remains in close proximity to the tRNA gene in an inactive state to quickly resume transcription upon changing to more favorable conditions. TFIIIC could act as a transient barrier or as an active repressor (or activator) by recruitment of repressive (or active) marks.

In summary, the observations that TFIIIC occupancy on tRNA genes and the interaction with TFIIIB increase upon nutrient perturbation suggest a dual state for TFIIIC (Figure 3). On the one hand, TFIIIC acts as a stimulator of TFIIIB recruitment and RNAPIII transcription initiation. On the other hand, TFIIIC could act as a physical barrier for RNAPIII transcription by its increased occupancy on the tRNA gene. Alternatively, it has been suggested that TFIIIC can act as a placeholder by occupying the tRNA gene body to maintain a nucleosome-free region [46]. Increased TFIIIC occupancy and interaction with TFIIIB in repressive conditions could reflect the preservation of ‘readiness,’ which supports rapid re-initiation of RNAPIII transcription once shifted to more favorable conditions. In line with this model, TFIIIB occupancy is only mildly reduced in repressed conditions [40,41,43], suggesting that RNAPIII-transcribed genes retain their GTFs to quickly resume transcription upon changing conditions. How this process of RNAPIII re-initiation, and thus TFIIIC displacement from the tRNA gene, is regulated remains unclear.

## Roles for TFIIIC beyond RNAPIII transcription

The dynamics of TFIIIC observed upon nutrient perturbation suggest that TFIIIC occupancy at tRNA genes is independent of active RNAPIII transcription or TFIIIB occupancy, at least in yeast cells [39,41,42]. Similar evidence was found in mammalian cells [47]. Here, tumor suppressor p53 was shown to repress transcription by RNAPIII through the disruption of Brf1 interaction with TFIIIC (TFIIIC110) and RNAPIII. Upon p53 induction, TFIIIB occupancy (TBP, Brf1, and Bdp1) decreases, whereas TFIIIC occupancy (TFIIIC63 and TFIIIC102) is unaffected. On the contrary, p53-/- fibroblasts show increased occupancy of TFIIIB (TBP) and RNAPIII, while TFIIIC (TFIIIC110) is unaffected [47]. If the binding of TFIIIC is independent of active transcription, TFIIIC binding to DNA may simply be driven by recognition of conserved DNA-binding domains such as the A- and B-boxes. In line with this is the observed occupancy of TFIIIC at so-called ETC (extra TFIIIC) sites, genomic loci that contain conserved (extended) B-box motifs but lack binding of TFIIIB and RNAPIII [48–52]. Despite identifying these loci and their chromatin barrier and insulator functions—independent of proper peripheral localization [53]—their exact role remains to be determined. While only eight ETC sites are identified in yeast, the human genome contains over 1800 TFIIIC-binding sites with extended B-box motifs and lack of TFIIIB and RNAPIII [51,54]. TFIIIC bound at these transcription-independent genomic loci is linked to a putative role in insulation and genome organization in yeast (also reviewed in [49]). tRNA genes have also been shown to have insulator activity to prevent the spread of repressive heterochromatin. This activity depends on the binding of TFIIIC [55,56]. Similarly, ETC sites have been shown to act as heterochromatin barriers that also depend on TFIIIC [56,57]. In addition to their role in stopping heterochromatin spreading, tRNA genes affect genome organization and activity by negatively affecting enhancer-promoter contacts [58–60]. Enhancer-blocking activity is mediated by CTCF and cohesin [61], and human tRNA genes are also described to act as enhancer-blocking insulators in a TFIIIC-dependent manner [60]. At ETC sites, a correlation was observed between CTCF and TFIIIC occupancy [51]. This correlation was confirmed in the mouse genome [58] and in breast cancer cells, TFIIIC co-immunoprecipitates with CTCF to possibly form long-range interactions with CTCF, mediating DNA looping [59]. The interaction between TFIIIC and CTCF seems to be context-dependent as it is observed in serum-starved breast cancer cells but disappears upon serum stimulation [59]. In addition, in mESCs or HEK293 cells, co-IP studies have not been able to validate an interaction between CTCF and TFIIIC [62]. These conflicting results provide incentive for additional studies to further our understanding of TFIIIC beyond RNAPIII transcription regulation.

**Perspectives**

- RNA polymerase III (RNAPIII)-specific general transcription factors (GTFs) are central hubs that initiate and regulate RNAPIII transcription from type I, II, and III promoters. Structural biology has recently provided important insights into the way these GTFs recognize type I, II, and III promoter sequences and recruit RNAPIII.
- GTF TFIIIC assumes multiple functions. TFIIIC participates in RNAP PIC formation on type I and type II promoters, but also recruits TFIIIB, and under nutrient deprivation acts as a transient barrier or as an active repressor of RNAPIII transcription. TFIIIC (without TFIIIB and RNAPIII) also binds to ETC sites presumably as an insulator.
- Under repressive conditions TFIIIB and TFIIIC remain partially bound or stay in the vicinity of type II promoters consistent with their roles in ‘facilitated re-initiation’ where RNAPIII re-engages with the same promoter using prebound GTFs by a so far unknown mechanism.

## Abbreviations

Bdp1: B-double prime 1
Brf1: TFIIB-related factor 1
DSE: distal sequence element
ETC: extra TFIIIC
GTFs: general transcription factors
IE: intermediate element
PIC: pre-initiation complex
PSE: proximal sequence element
RNAPIII: RNA polymerase III
SNAPc: snRNA activating protein complex
TBP: TATA-binding protein
TSS: transcription start site
ZF: zinc fingers
co-IP: co-immunoprecipitation
mTOR: mammalian target of rapamycin
τIR: interaction region
